# Advancements, Challenges, and Future Perspectives of Soybean-Integrated Pest Management, Emphasizing the Adoption of Biological Control by the Major Global Producers

**DOI:** 10.3390/plants15030366

**Published:** 2026-01-24

**Authors:** Adeney de F. Bueno, William W. Hoback, Yelitza C. Colmenarez, Ivair Valmorbida, Weidson P. Sutil, Lian-Sheng Zang, Renato J. Horikoshi

**Affiliations:** 1 Embrapa Soja, Rodovia Carlos João Strass, s/n, Distrito de Warta, P.O. Box 4006, Londrina 86085-981, Brazil; 2 Department of Entomology and Plant Pathology, Oklahoma State University, Stillwater, OK 74078, USA; whoback@okstate.edu; 3 CABI-UNESP-FEPAF, Rua José Barbosa de Barros, 1780, Botucatu 18610-307, Brazil; y.colmenarez@cabi.org; 4 Division of Plant Science and Technology, University of Missouri, Columbia, MO 65201, USA; ivairvalmorbida@missouri.edu; 5 Biological Sciences Sector, Department of Biology, Universidade Federal do Paraná, Jardim das Américas, Curitiba 80035-050, Brazil; plauter80@gmail.com; 6 State Key Laboratory of Green Pesticides, Guizhou University, Guiyang 550025, China; lszang@gzu.edu.cn; 7 Bayer Crop Science, Santa Cruz das Palmeiras 13650-000, Brazil; renato.horikoshi@bayer.com

**Keywords:** IPM, sustainability, *Bt* cultivars, economic thresholds

## Abstract

Soybean, *Glycine max* (L.) Merrill, is usually grown on a large scale, with pest control based on chemical insecticides. However, the overuse of chemicals has led to several adverse effects requiring more sustainable approaches to pest control. Results from Integrated Pest Management (IPM) employed on Brazilian soybean farms indicate that adopters of the technology have reduced insecticide use by approximately 50% relative to non-adopters, with yields comparable to or slightly higher than those of non-adopters. This reduction can be explained not only by the widespread use of *Bt* soybean cultivars across the country but also by the adoption of economic thresholds (ETs) in a whole Soybean-IPM package, which has reduced insecticide use. However, low refuge compliance has led to the first cases of pest resistance to Cry1Ac, thereby leading to the return of overreliance on chemical control and posing additional challenges for IPM practitioners. The recent global agenda for decarbonized agriculture might help to support the adoption of IPM since less chemical insecticides sprayed over the crops reduces CO_2_-equivalent emissions from its application. In addition, consumers’ demand for less pesticide use in food production has favored the increased use of bio-inputs in agriculture, helping mitigate overdependence of agriculture on chemical inputs to preserve yields. Despite the challenges of adopting IPM discussed in this review, the best way to protect soybean yield and preserve the environment remains as IPM, integrating plant resistance (including *Bt* cultivars), ETs, scouting procedures, selective insecticides, biological control, and other sustainable tools, which help sustain environmental quality in an ecological and economical manner. Soon, those tools will include RNAi, CRISPR-based control strategies, among other sustainable alternatives intensively researched around the world.

## 1. Introduction

Soybean, *Glycine max* (L.) Merrill, is one of the most important crops around the world [1]. It supplies 60% of vegetable protein and roughly 30% of the world’s edible oil [2]. Production reached 396 million metric tons in the 2023/2024 season [3] and will continue to grow because of the increasing global demand for food and biodiesel [4]. However, soybean yields can be severely impacted by outbreaks of a variety of pest species, especially from caterpillars (Lepidoptera) and hemipterans [5]. These pests cause global losses of at least 29% when not properly managed [6,7]. In Brazil, the world leader in soy production, soybean losses from pests have reached 4.31 million tons each year [8].

Although chemical insecticides remain effective against pests [9] and play an important role in managing soybean pests [3,10], the overdependency on them raises concerns about their lasting impact on the environment and human health [11,12]. The overuse of chemicals can trigger adverse side effects, including outbreaks of secondary pests [13], selection of resistant pests [14], and detrimentally impact pollinators [15] and biocontrol agents [16], in addition to other negative impacts [17]. Consequently, reducing the use of chemical insecticides in agriculture is increasingly demanded [18] and an important goal of public policies around the world [19].

Integrated Pest Management (IPM) is an efficient strategy to control pests in agriculture [20]. IPM improves safety to non-target organisms [21] and final consumers [22], while also increasing farmers’ profits [10]. As a science-based pest management approach, Soybean-IPM aims to use a combination of strategies, including the planting of resistance cultivars (including *Bt* soybean), cultivation measures, pest identification and scouting, adoption of biological control, and use of less harmful chemicals only when necessary (based on established ETs), to manage pests while minimizing risks to people, non-target organisms, property, and the environment [23].

**Figure 1:**
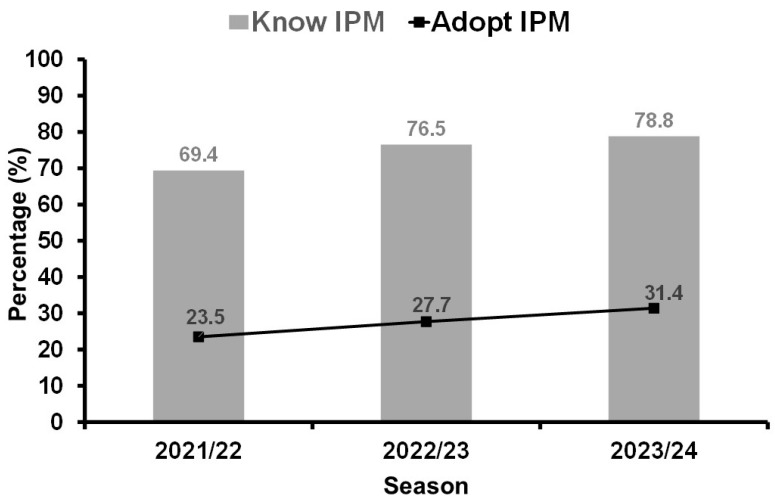
Percentage of soybean farmers in the state of Paraná, Brazil, who know the IPM principles (gray bars) and the percentage who have adopted IPM (black line) in their fields. Adapted from [24,25,26].

Despite the known benefits provided by its adoption, IPM has not been implemented with the necessary intensity by many growers [13]. Despite the increasing knowledge and adoption of Soybean-IPM, there are still a large number of farmers who do not know about IPM (21.2%, 2023/24) and even a greater number of farmers who, despite knowing the strategies, do not practice Soybean-IPM (68.6%, 2023/24) (Figure 1). For instance, in the state of Paraná, Brazil, while 78.8% of the soybean farmers declare knowing how to practice Soybean-IPM, only 31.4% of them have adopted the strategy. Nevertheless, Soybean-IPM is considered the most successful IPM program developed in Brazil, reducing the amount of insecticide used to control insect pests by about 50% [10].

**Figure 2:**
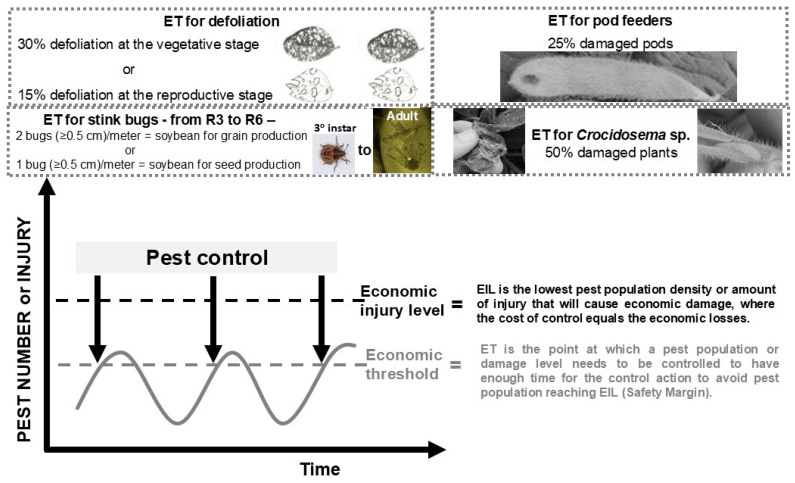
Economic injury levels (EILs) in relation to the most important economic thresholds (ETs) for soybean pests recommended in Brazil. Adapted from [27,28,29,30].

Since the 2013/14 growing season, the Soybean-IPM program in Paraná has been reinforced by cooperation between federal (represented by the research institution, Embrapa Soja) and state (represented by the extension service and research institution, Paraná Rural Development Institute—IDR) governments. Furthermore, several farmers allowed their soybean fields to be used to demonstrate the benefits of adopting sustainable Soybean-IPM. Those soybean fields have been called Unit of Technological Reference, which have been monitored by the IDR-Paraná extension program throughout the whole soybean growing season. An extensionist was responsible for one or more weekly pest samplings (scouting) in the field and for quantifying pests per meter and defoliation. Insecticides were applied only when economic thresholds (ETs) (Figure 2) were reached or surpassed [10]. Simultaneously, a survey was carried out, with the aid of a questionnaire, with farmers not assisted by the Soybean-IPM program, to quantify the number and time of chemical applications. This data was used to compare non-assisted and assisted farmers and evaluate the results and challenges of adopting Soybean-IPM in the state, as further discussed in the following sections.

## 2. Soybean-IPM: A Successful Case Study from the State of Paraná, Brazil

Comparing the results from farms that adopted Soybean-IPM with those that did not, across eleven soybean seasons, adopters reduced the average number of insecticide applications by 52.8% (varying from 43.3% in 2019/20 to 69.2% in 2021/22 seasons) compared with farmers who did not adopt IPM. This reduction lowered pest control costs by 51.6%, which is equivalent to 117 kg of soybean per hectare (ha). IPM not only reduced pest control costs but also slightly increased average yield by 93.8 kg/ha (2.8%), resulting in an average profit increase of 210.7 kg/ha (Table 1). Thus, the adoption of Soybean-IPM increased farmers’ profit in addition to being a more sustainable and efficient pest management [10,31,32,33,34,35]. These results are not surprising as the profitability of IPM adoption has been consistently reported in the scientific literature [36]. A reported economic return from employing IPM in soybean ranged from USD 0.6 to USD 2.6 billion in 2005 for USA farmers [37]. Similar positive economic results have also been reported for Soybean-IPM adopters in Brazil [38], Argentina [5], India [39], and Indonesia [40].

Not only is Soybean-IPM more profitable to farmers, but it is also safer for the environment, preserving biological control agents and pollinators. The number of days from sowing to the first insecticide application increased approximately 37% from 46.9 days (non-adopters) to 73.4 days for the Soybean-IPM adopters (Table 1). Delaying insecticide application by 26.5 days is an important strategy to conserve predators, parasitoids, pollinators, and other beneficials, which provide critical ecosystem services [10].

Despite not being universally adopted (Figure 1), Soybean-IPM definitely shows success in Brazil [41], leading to a significant reduction in insecticide sprays compared with non-adopters of the technology [10]. The reduction in traditional chemical insecticides used in soybean has been a result not only of using ETs as a basis for IPM decisions [42], but also as a consequence of the increasing use of biological control agents and adoption of *Bt* soybean cultivars in Brazil. These are further discussed in the following sections of this review.

**Table 1:** Results from 11 years of adoption of Soybean-IPM ^1^ in the state of Paraná, Brazil, compared with farmers who did not adopt IPM. Adapted from [24,31,32,33,34,35,43,44,45].

Soybean Season	Number of Fields		Number of Sprays (Insecticides)		Days Until First Insecticide Spray		Pest Control Costs ^2^ (kg/ha)		Yield (kg/ha)		Increased Profits ^2,3^ (kg/ha)
	Adopter	Non-Adopter	Adopter	Non-Adopter	Adopter	Non-Adopter	Adopter	Non-Adopter	Adopter	Non-Adopter	
2013/14	46	333	2.3	5.0	57.5	33.0	144	302	2952	2922	186
2014/15	106	330	2.1	4.7	66.0	34.0	120	300	3612	3516	276
2015/16	123	314	2.1	3.8	66.8	36.0	120	240	3426	3282	264
2016/17	141	390	2.0	3.7	70.8	40.5	138	246	3870	3828	150
2017/18	196	615	1.5	3.4	78.7	43.6	138	324	3702	3630	258
2018/19	241	773	1.7	3.4	74.0	40.3	126	246	3006	2916	210
2019/20	255	553	1.7	3.0	75.0	56.0	108	186	3864	3804	138
2020/21	191	518	1.7	3.4	76.0	59.0	60	120	3654	3618	96
2021/22	175	522	0.8	2.6	85.0	57.0	36	96	1752	1740	72
2022/23	150	443	1.0	3.0	86.0	61.0	54	156	4128	4002	228
2023/24	138	543	1.7	3.3	72.0	56.0	162	276	3552	3228	438
Average	160.8	484.9	1.7	3.6	73.4	46.9	109.6	226.6	3410.7	3316.9	210.7

^1^ IPM program where public consultants (from IDR—Paraná) sampled pests during the seasons and made all the decisions about IPM in the fields of selected farmers. At the end of the season, the results of IPM fields were compared with fields of non-adopters of IPM throughout the state. ^2^ Pest control costs and increased profits from adopting IPM compared with non-adoption were transformed into the equivalent of the value of kilograms of soybean for each season to avoid any depreciation of the currency due to possible effects of inflation. ^3^ Increase profits = (Yield of Adopters − Yield of Non-Adopters) + (Pest Control Costs of Non-Adopters − Pest Control Costs of Adopters).

## 3. Use of Economic Thresholds (ETs) in Soybean-IPM

**Figure 3:**
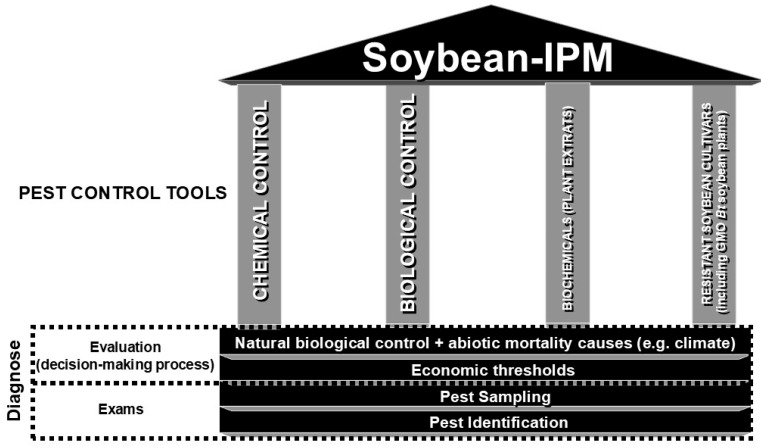
Soybean-IPM structure, sustained by the association of different pest management tools and based on diagnosis.

Pest monitoring (insect sampling and identification) and decision-making, comparing pest populations with ETs, are the basis of Soybean-IPM (Figure 3) and crucial to its success [46]. ETs are based on the premise that cultivated plants can tolerate certain levels of injury without economically significant yield reductions [47]; therefore, not all herbivorous insects will become pests and/or require control (Figure 2) [48,49]. In this context, a decision to control any pest species in soybean should only be made when the pest population is equal to or greater than previously established ETs (Figure 2, Table 2) or is expected to surpass those levels within hours or a few days [42].

Around the world, the established ETs for soybean pests slightly differ because of variations in crop value; different adopted cultivars, pest control costs, pest species occurring, and local environmental conditions; and the availability and effectiveness of control technologies [57]. For instance, different ETs for defoliators (Lepidoptera) and stink bugs (Hemiptera: Pentatomidae) have been reported between Brazil and the USA, which are the first and second global soybean producers, respectively. In Brazil, the ET for defoliators is 30% defoliation (in the vegetative stage) or 15% defoliation (in the reproductive stage) [42]; however, in the USA, this ET is 35% defoliation at the vegetative stage and 20% at the reproductive stage [53]. ETs can also vary between regions in the same country [53]. In the USA, the ET for defoliation ranges from 40% to 30% defoliation during vegetative stages and from 25% to 15% defoliation during reproductive stages in different growing areas of the country (Table 2).

ETs for stink bugs vary less than for defoliators. The recommended ET for stink bugs in soybean is two insects larger than 0.5 cm (including nymphs from third instar to adults) per row meter if the fields are intended for grain production or only one bug if the field is used for seed production in Brazil [42]. In the USA, the ET is three bugs larger than 0.6 cm per row meter if a beat cloth is used as the sampling method or ten bugs per 25 sweeps [53] (Table 2).

Despite the remaining challenges, ETs are generally well established for the most important pests of soybean (Table 2). Some occasional or sporadic soybean pests, such as mites, thrips, and whiteflies, require more research to precisely establish ETs [42]. For instance [58], a study of different ETs for whitefly control in soybean recorded that yield was only reduced when whitefly outbreaks were extensive enough to trigger the growth of sooty mold, *Capnodium*, on the leaves. However, the growth of sooty mold on whitefly-infested soybean differs depending not only on pest infestation but also on soybean cultivars [54], making it difficult to establish a number of insects per foliar area as an ET.

Despite these challenges, ref. [52] proposed an ET for whitefly on soybean of 1.5 insects per leaflet. This very conservative ET is seven times lower than the ET of whitefly on cotton [59]. However, the yield results by [52] of 18.73 g plant^−1^, from treatment receiving seven insecticide sprays and resulting in 0.35 whiteflies per trifoliate (0.1 whitefly per leaflet), were statistically equal to results from the control (without any insecticide spray), with 27.35 whiteflies per trifoliate (9.1 whitefly per leaflet). These results contradict realistic ET and principles of IPM [47,60]. Moreover, the size of a soybean leaflet can vary considerably depending on the plant’s developmental stages, cultivar, and environmental conditions [61], reinforcing the need for further studies with whiteflies to determine ET.

Similarly, ETs for mites also remain understudied [13]. An ET of approximately 21 individuals per soybean leaflet of *Tetranychus cucurbitacearum* (Sayed) was proposed by [56]. However, as previously noted, the size of a soybean leaflet varies [61]. Later, an economic injury level (EIL) for *Tetranychus urticae* in soybean based on population density was determined as one *T. urticae* per cm^2^ of leaf area, considering the control cost of USD 20.00 ha^−1^ and the soybean crop value of USD 350.00 Mg^−1^ [62]. Nevertheless, the ET, which is the direct tool used by farmers to make decisions, is still unclear.

Thrips are common in soybean despite rarely causing direct economic damage, although dry and hot weather can lead to high populations. The EIL for thrips in soybean was estimated to be between 4.53 and 3.43 thrips per plastic beating tray (40 × 25 × 3 cm) placed beneath the plant’s apex while the branch bearing the apical foliage is struck sharply [63]; however, no ET has been proposed.

Despite the differences in recommendations and the remaining challenges posed by more sporadically occurring pest species, the principle behind ETs is to avoid preventive insecticide applications [10]. Globally, threshold-based programs reduced overall insecticide applications by 44% and associated costs by 40%, without compromising pest control or yield compared with calendar-based programs [64], making them economically advantageous for farmers and ecologically beneficial to the environment. Nevertheless, farmers and pest managers often question these thresholds and apply insecticides when pest densities are well below recommended ETs [13,53].

Among the range of reasons for such low adoption of ETs, two crucial challenges are that (1) farmers usually fear facing significant yield loss without spraying insecticides, consequently resulting in the refusal to fully adopt ETs and, especially, (2) the amount of work required for pest monitoring [13]. Assessing soybean pest numbers or their injuries is required for ET use but is frequently perceived as too time-consuming [13,50]. The shortage of farm labor is a reality for soybean farmers due to urbanization and the migration of people from rural to urban centers [65]. Furthermore, the lack of workers, inadequate training and capacity-building for IPM practitioners, and insufficient attention to IPM adoption are challenges for ET adoption [66]. After training, soybean farmers increase their adoption of ET, consequently decreasing pesticide use [67].

## 4. Use of *Bt* Cultivars in Soybean-IPM

In Brazil, the Soybean-IPM heavily utilizes prevention strategies [9]. Before sowing the soybean field, choosing resistant cultivars plays an important role in the success of IPM [68] as an economically, ecologically, and environmentally safe decision [69]. Resistant plants suffer less damage from pests than susceptible counterparts, consequently eliminating or reducing the need for insecticides to keep pest populations below economic thresholds [70]. Genetically modified (GM) plants, resistant to insects, represent a more recent insect pest control method for IPM programs in various agroecosystems [71].

**Figure 4:**
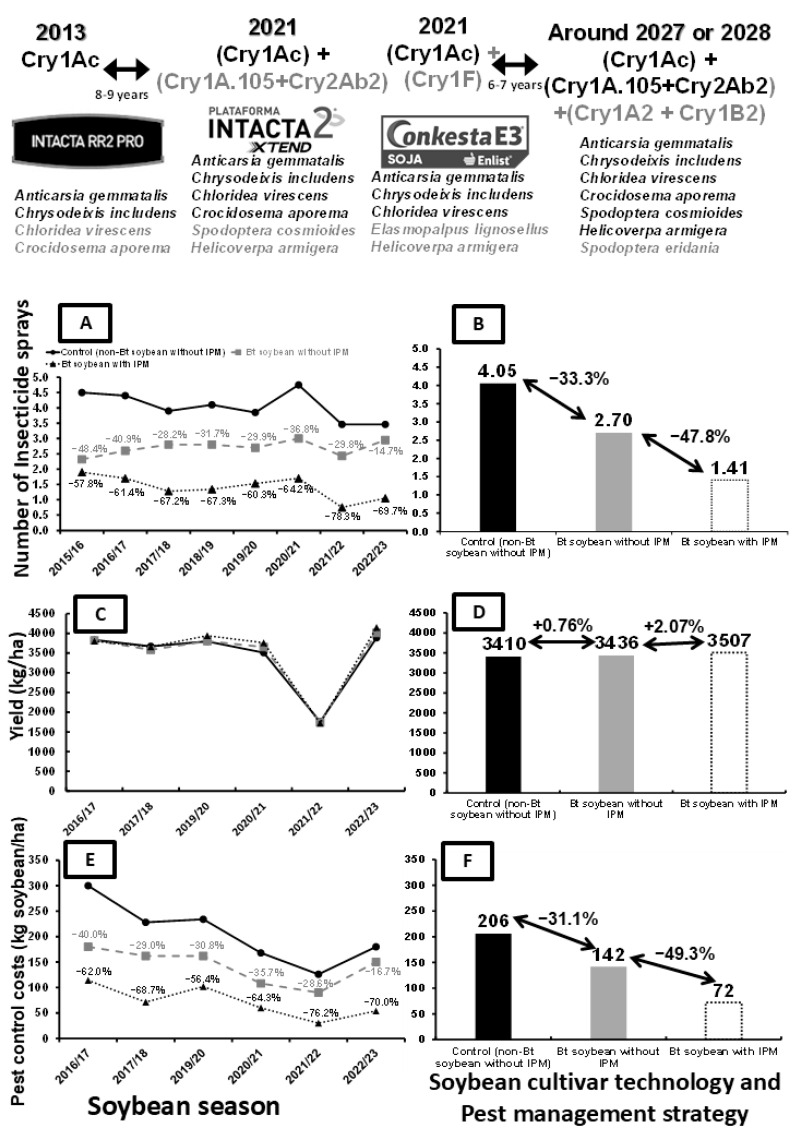
Results from the adoption of *Bt* soybean with and without the adoption of IPM in the State of Paraná, Brazil, during eight crop seasons from 2016/17 to 2022/23. (**A**) Insecticide spray per season and (**B**) mean spray of insecticides of different pest management technologies. (**C**) Yield over the seasons and (**D**) mean yield according to the adopted pest management technology. (**E**) Pest control costs over the seasons and (**F**) mean pest control cost according to the adopted pest management technology. Adapted from [73].

Since its first commercial release in 1995, crops genetically transformed with the addition of Cry proteins from *Bacillus thuringiensis* (*Bt*) have increasingly been part of the agricultural landscape and an important tool in IPM. GM plants have been widely adopted by at least 26 different countries spread worldwide [72]. As in other crops, Soybean-IPM has been transformed by the introduction of the *Bt* soybean technology expressing Cry1Ac (event MON 87701) protein (*Bt*) at high levels due to its high efficacy and simplicity of adoption [73]. Initially (2013), growers adopted the first generation of this technology (expressing only Cry1Ac) and later (2021) enjoyed the addition of the second generation (expressing Cry1Ac + Cry1A.105 + Cry2Ab2 for Intacta 2 Xtend and Cry1Ac + Cry1F for Conkesta) (Figure 4). The use of *Bt* soybean has been especially high in South America, particularly in Brazil and Argentina, the first- and third-largest world soybean producers, respectively (Table 3). By the fifth year of *Bt* soybean adoption in South America, *Bt* soybean was cultivated over an area of 73.6 million hectares, generating an increased income of USD 7.64 billion for farmers [74] and reducing pesticide use by approximately 10.44 million kg. This reduction in pesticide use and field operations contributed indirectly to lower greenhouse gas (GHG) emissions, primarily by decreasing energy demand associated with pesticide manufacturing, transport, and application. Estimates indicate that this mitigation effect is equivalent to removing approximately 3.3 million cars from the roads in terms of CO_2_-equivalent emissions [74,75].

The reduction in insecticide applications in soybean fields has occurred both with and without the adoption of IPM when adopting *Bt* cultivars (Figure 4A). The adoption of just *Bt* soybean (at farms not adopting IPM) reduced insecticide applications from 48.4% (2015/16 crop season) to 14.7% (2022/23 crop season). When *Bt* soybean was adopted as a pest management strategy within the IPM framework, especially associated with pest sampling and insecticide application only when the pest population reaches or surpasses ET, insecticide use was reduced even further. Reductions from 57.8% (2015/16 crop season) to 78.3% (2021/22 crop season) were recorded (Figure 4A). On average of all seasons (Figure 4B), an insecticide reduction of 33.3% with adoption of *Bt* cultivars without IPM is noted compared with the control, with this reduction increasing to 65.2% with the adoption of *Bt* cultivars with IPM compared with the control. These results reinforce the importance of the adoption of *Bt* cultivars and IPM to further reduce the use of insecticides, highlighting the additive effect of *Bt* technology combined with pest monitoring and economic threshold-based decision-making adoption.

The reduction in insecticides associated with *Bt* crops has been reported in the literature in other crop systems. In the USA, insecticide applied in maize, *Zea mays*, fields decreased 75% with the adoption of *Bt* varieties, falling from 0.2 kg/ha in 1998 to about 0.05 kg/ha in 2011, when the adoption of *Bt* varieties exceeded 80% of the maize cultivated in the country [77]. This lower use of insecticide has benefited the conservation of natural biological control agents in different agroecosystems [72], including cotton, corn, potato, rice, and eggplant [78], as well as soybean [73]. Because *Bt* has negligible effects on non-target organisms [79], it is regarded as a safer choice than chemical insecticides [80] to manage target pests.

Despite the recorded benefits of *Bt* soybean over insecticides, some negative effects have been recorded due to the over adoption of the technology (higher than the 80% limit), lacking compliance for refuges (adoption of at least 20% the field with non-*Bt* cultivars) as Insect Resistance Management (IRM) [73]. Refuge areas (non-*Bt* cultivars) managed with IPM are essential to produce insects susceptible to *Bt*, preserving the efficacy of *Bt* cultivars. Consequently, the resurgence of target pests associated with the populations resistant to the *Bt* toxins has been reported [81,82], including for *Rachiplusia nu* [83] and *Crocidosema* sp. [84] in Brazilian soybean fields.

Outbreaks of secondary pests in soybean have also been associated with the adoption of *Bt* cultivars [85,86]. With the reduction in the insecticide load in the crop, *Spodoptera* spp., which have been known for tolerance to commercial *Bt* cultivars and were previously controlled by insecticides applied for other lepidopterans, have survived and been reported as attacking soybean leaves and plant reproductive structures, potentially reducing yield [85]. Despite these negative effects, which are also similarly reported for conventional insecticides [13], the potential conservation of the biocontrol diversity in the *Bt* soybean agroecosystem are valuable. Reductions in insecticide use due to the adoption of *Bt* cultivars have been reported in soybean, not only in Brazil [73] but also in Argentina [87] and Uruguay [88], which is certainly helpful to conserve natural biological control and, therefore, mitigate pest outbreaks in the fields [10].

Importantly, the reduction in conventional insecticide use due to the adoption of *Bt* soybean does not cause measurable yield reduction (Figure 4C). In fact, *Bt* soybean fields had a higher yield on average than non-*Bt* fields (Figure 4D), likely as a result of better lepidopteran control. In addition, fields planted with *Bt* cultivars had lower pest control costs (Figure 4E,F). Pest control costs (transformed to their equivalent in value of kg soybean/ha in each crop season) were between 16.7% (2022/23 crop season) and 40.0% (2015/16 crop season) lower for *Bt* fields without IPM than non-*Bt* fields without IPM. The adoption of *Bt* soybean in the IPM framework reduced pest control costs even more, from 56.4% (2019/20 crop season) to 76.2% (2021/22 crop season), compared with control fields (non-*Bt* without IPM) (Figure 4E). This is an average reduction in costs equal to 64 kg/ha (31.1%) (*Bt* soybean with IPM adoption) compared with the control (non-*Bt* without IPM fields) (Figure 4F). The combined impacts of the adoption of *Bt* cultivars on reducing production costs associated with higher yields (consequences of better pest control) are clearly resulting in significant increases in profits. A national survey carried out from 1998 to 2017 in Brazil, including both soybean *Bt* and herbicide resistance traits, found that farmers planting GM soybean had 26% higher profits than those using conventional cultivars [89].

## 5. Role of Biological Control in Soybean-IPM

The intensification of agriculture has been required to ensure food security for an increasing global population [90]. However, this intensification also provides more food to pests, favoring their outbreaks [91]. This sequence of events demands more and new crop protection options because the pressure to decrease insecticides has also increased [18]. These apparently conflicting demands (intensification of food production versus reduction in insecticide use) increase the importance of safer alternatives such as biological control [92,93,94]. Consequently, the augmentative biological control market has been growing globally, and it is expected to surpass USD 10 billion in 2027 [95].

Biopesticides are increasingly recognized for their effectiveness in controlling pests [96,97]. These biocontrol agents play an essential role in IPM [10] and can be applied either alone or in combination with synthetic selective pesticides [98]. This approach enhances control efficacy, reduces environmental risks, and the exposure of farmers and consumers to synthetic pesticides [95]. Moreover, biopesticides help manage not only pest populations but also their resistance, as biopesticides allow the rotation and diversification of control tactics with distinct modes of action, reducing selection pressure associated with repeated use of the same chemical groups [99], which is a major problem for soybean production [14,100].

In addition to augmentative biological control, conservation biological control is important for the success of Soybean-IPM, despite the potential of natural biological control to help maintain soybean pests below ETs being frequently underestimated [98]. For instance, common hemipteran predators found in soybean, such as those from the genus *Geocoris* and *Nabis*, have predation capacities of consuming 9 and 21 Lepidoptera eggs per day, respectively [101]. The soybean agroecosystem is rich not only in hemipteran predators but also in coleopterans [102] and a great number of other biocontrol agents, including parasitoids [103] and entomopathogens [104].

Larvae of *Callida* spp. consume around 65.6 velvetbean caterpillars, *Anticarsia gemmatalis* (Hübner) [105], while the egg parasitoid *Telenomus podisi* can parasitize about 100 eggs of the stink bugs *Euschistus heros* (Fabricius) [106] and *Diceraeus melacanthus* Dallas (Hemiptera: Pentatomidae) [107] during its lifespan. A recent study from China demonstrated that *Trichogramma leucaniae* reared on eggs of the Eri silkworm *Samia ricini* Willian Jones can parasitize, in a 24 h period, 27–48 eggs of the soybean pod borer *Leguminivora glycinivorella* (Matsumura), which is a critical pest for soybean farmers in the county [108].

The potential of conserving those biocontrol agents in the soybean agroecosystem was illustrated by [109], who recorded the mortality of *Helicoverpa armigera* (*Hübner*) (*Lepidoptera*: *Noctuidae*). When the pest was first reported in Brazil in the 2013/14 crop season, they suffered 70.2% of natural mortality [10]. In modern agriculture, biological control is crucial to the success of IPM, and IPM is essential for providing a more stable and favorable environment that enables biocontrol agents to express their full potential in pest control and succeed [110].

## 6. Opportunities for Increasing the Adoption of Soybean-IPM

As previously discussed in this review, despite successful examples of Soybean-IPM in major world producers and numerous benefits from its adoption, barriers remain in increasing its acceptance and further adoption. Key barriers include inadequate training and a lack of technical support for farmers; shortages of IPM specialists and extension agents; and a lack of simultaneously managing recommendations for multiple pest species, the most common situation faced by soybean farmers [13]. To increase Soybean-IPM adoption, strategies must address the whole soybean pest complex occurring in each region, and ideally offer simple, effective solutions that are common with insecticide sprays [111].

While earlier IPM models were restricted to ecological and economic aspects based on chemical control, newer IPM models include management, business, and sustainability, emphasizing the importance of research and outreach as well as various social factors that influence the market of IPM products [20]. Moreover, governmental banning of several harmful chemical insecticides and the advent of more selective options further increase the potential for integrating chemical and biological control [112]. Finally, the more recent agenda focused on reducing carbon emissions from agriculture has been intensified [113]. The significant greenhouse gas (GHG) emissions from agriculture, including non-carbon gases, require immediate action to meet emission reduction goals and address global climate change [114]. Federal actions, along with broader societal efforts, focus on mitigating non-CO_2_ emissions like methane and scaling up CO_2_ removal initiatives [115,116]. This new global arena has encouraged the adoption and renewed interest in Soybean-IPM principles, giving the technology its momentum.

Reduction in insecticide use due to Soybean-IPM adoption is directly linked with a proportional decrease in the diesel used to apply such chemicals, as well as a reduction in CO_2_ emissions related to the mitigation of this operation for pest control in the field. For instance, Soybean-IPM adoption in Brazil in the 2021/22 crop season reduced emissions by 6,025.82 kg CO_2_ equation for every 100 hectares of soybean [10]. Similar benefits have been reported for the overall adoption of GM soybean in Brazil, saving 79.2 million liters of diesel from 1998 to 2017. This amount of fuel is enough to power around 53,000 cars for one year [108].

Certification protocols of the Low Carbon Soybean Program (LCSP) initiative, led by EMBRAPA SOJA in Brazil, can also work as incentives to soybean farmers without depending on the federal government. This program aims to add value to soybean by certifying its sustainability, with the LCSP anticipating a reduction of approximately 30% in emissions per ton of soybean through methods like no-till farming and inoculants to reduce nitrogen fertilizer use [117,118]. Thus, Soybean-IPM certainly fits into the scope of this initiative.

Increased federal actions that favor the adoption of Soybean-IPM could include financial incentives to practitioners of the technology. For instance, in Brazil, federal government programs offer credit lines with subsidized interest rates and special conditions for other sustainable practices in agriculture, including the use of technologies that promote nutrient efficiency, such as the inoculation of soybean seeds with *Bradyrhizobium* and *Azospirillum*. These subsidized credit lines have made inoculation practices more financially attractive, increasing adoption to 85% of the soybean cultivated area in the 2022/2023 season, using around 8.4 billion liters or kilograms of inoculants [118]. Similar support for Soybean-IPM would certainly increase its adoption.

## 7. Final Considerations and Future Perspectives of Soybean-IPM

There is increased interest in cultivating soybeans in a productive and ecologically sound manner that yields healthy food while protecting environmental integrity for future generations. Not all technologies that increase productivity are free of negative impacts on long-term sustainability. For these reasons, there is a need to develop approaches that are stable, resilient, and sustainable as well as productive and profitable for farmers. Resistance to insecticides, herbicides, and other pesticides has led to higher application rates of chemical insecticides, greater crop losses, and increased production costs for soybean farmers. In addition, the increasing use of pesticides is closely linked to elevated health risks for farmers, farm workers, rural populations, and final consumers. Overusing pesticides brings negative effects on soil health, water quality, and wildlife habitats. The non-market costs of insecticide negative impacts are only estimable globally and impose a significant burden.

Various pest control strategies, such as biotechnology, biological control, and insecticides, have been recognized as important breakthroughs in food production. Nevertheless, they must be used within IPM to achieve long-term efficacy. In this context, biological control, the rational use of more selective insecticides, the adoption of ETs for control decisions, and the cultivation of *Bt* soybean have been key elements of the success of Soybean-IPM. The IPM concept integrates a wide array of alternative approaches, including biopesticides (macro- and microorganisms), botanical pesticides (essential oils, plant extracts), and genetic pest management methods, such as the sterile insect technique, genome-editing tools (e.g., CRISPR-Cas9, RNAi), and marker-assisted selection) as well as any other sustainable alternative focused on the multiple pest species scenario usually faced by farmers. Proposing a simple, efficient, straightforward combination of solutions will make this technology more acceptable to farmers.

Future trends of Soybean-IPM include (1) maximizing the efficacy of biocontrol agents; (2) the development and use of genetic tools, such as DNA and CRISPR-Cas9 technologies; (3) improvement in plant resistance, including the development of newer GMs and genetically edited cultivars; (4) nanoformulations and encapsulations for microbiological and botanical insecticides, including water–oil emulsion encapsulation for *Bacillus thuringiensis* to improve its stability, nanoformulations of *Bacillus* lipopeptides (Lps), and CRISPR-based technologies for managing pests. Mobile applications have become vital to the dissemination of IPM. Apps that use artificial intelligence to identify pest problems and recommend suitable interventions will help increase IPM efficacy and the adoption of Soybean-IPM.

## 8. Conclusions

Pests should not be exterminated; instead, they should be kept below EILs. In a more balanced agricultural system, no pest control method should achieve 100% pest mortality, as many farmers strive for. On the contrary, 100% control of a given pest is undesirable as it can lead to a decline in the natural biological control species due to the unavailability of prey or host to sustain their population, making the crop more susceptible to a reemergence of the pests and outbreaks of secondary pests. Thus, a critical and broader shift in farmers’ behavior is underway, and the most significant challenge is to achieve greater adoption of Soybean-IPM. GM soybean, such as *Bt* cultivars, has been an essential technology for the success of Soybean-IPM, combining better conservation of natural biological control with increased profits for farmers. Nevertheless, resistance must be managed effectively to avoid issues that could permanently impair the lifespan of *Bt* technology. In addition to biological control, Soybean-IPM is currently experiencing a positive phase. Nevertheless, greater efforts should be directed towards outreach, practitioner incentives, and technology research to avoid losing the favorable momentum for IPM implementation and success.

## Figures and Tables

**Table 2:** Economic thresholds (ETs) recommended for soybean pests.

Pests	ET(s)	References
*Aphis glycines*	273 ± 38 aphids/plant	[50]
*Bemisia tabaci*	(a) 1.5 insects per leaflet (b) Beginning of sooty mood formation	[51,52]
*Crocidosema* sp.	50% of damaged plants	[30]
Defoliators	(a) 30% defoliation (soybean in the vegetative stage)—Brazil, Illinois, Iowa, and North Dakota (USA) (b) 35% defoliation (soybean in the vegetative stage)—USA (c) 40% defoliation (soybean in the vegetative stage)—Michigan and Ohio (USA) (d) >40% defoliation (soybean in the vegetative stage)—Indiana (USA) or (e) 15% defoliation (soybean in the reproductive stage R1 to R6)—Brazil, Ohio and Michigan (USA) (f) >15% defoliation (soybean in the reproductive stage R1 to R6)—Indiana (USA) (g) 20% defoliation (soybean in the reproductive stage)—Illinois, Iowa, and North Dakota (USA)	[27,53]
*Helicoverpa zea*	3.5 caterpillars/m or sample cloth or 9 caterpillars/25 sweeps—USA	[54]
Heliothinae (*Helicoverpa* spp. and *Chloridea virescens*)	(a) 4 caterpillars/m or sample cloth (soybean in the vegetative stage)—Brazil or (b) 2 caterpillars/m or sample cloth (soybean in the reproductive stage)—Brazil	[24]
Pod feeders	25% damaged pods	[29]
*Spodoptera* spp.	10 caterpillars (≥1.5 cm)/m or sample cloth	[55]
Stink bugs	(a) 2 stink bugs (≥0.5 cm)/m or sample cloth (soybean for grain production)—Brazil (b) 3 stink bugs (≥0.6 cm)/m or sample cloth—USA (c) 9 stink bugs (≥0.6 cm)/25 sweeps—USA or (d) 1 stink bug (≥0.5 cm)/m or sample cloth (soybean for seed production)—Brazil	[28,52]
*Tetranychus cucurbitacearum*	21.23 mites/leaflet	[56]

**Table 3:** Adoption of *Bt* soybean cultivars in South American countries.

Country	Area (ha)	%	Year	Reference
Brazil	43.0 million	94	2023/24	[73]
Argentina	4.3 million	16.2	2018	[76]
Paraguay	1.7 million	6.4	2018	[76]
Uruguay	0.4 million	1.5	2018	[76]

## Data Availability

No new data was created in this review.
